# ‘They Say HIV is a Punishment from God or from Ancestors’: Cross-Cultural Adaptation and Psychometric Assessment of an HIV Stigma Scale for South African Adolescents Living with HIV (ALHIV-SS)

**DOI:** 10.1007/s12187-016-9428-5

**Published:** 2016-11-23

**Authors:** Marija Pantelic, Mark Boyes, Lucie Cluver, Mildred Thabeng

**Affiliations:** 1 Department of Social Policy and Intervention, University of Oxford, 32 Wellington Square, Oxford, OX1 2ER UK; 2 School of Psychology and Speech Pathology, Curtin University, GPO Box U1987, Perth, WA 6845 Australia; 3 Department of Psychiatry and Mental Health, University of Cape Town, Cape Town, South Africa; 4 Department of Social Work, University of South Africa, Pretoria, South Africa

**Keywords:** HIV/AIDS, Stigma, Adolescent, Psychometric assessment, Cognitive interviews

## Abstract

Sub-Saharan Africa is home to 90 % of the world’s adolescents living with HIV (ALHIV). HIV-stigma and the resultant fear of being identified as HIV-positive can compromise the survival of these youth by undermining anti-retroviral treatment initiation and adherence. To date, no HIV-stigma measures have been validated for use with ALHIV in Sub-Saharan Africa. This paper reports on a two-stage study in the Eastern Cape, South Africa. Firstly, we conducted a cross-cultural adaptation of an HIV stigma scale, previously used with US ALHIV. One-on-one semi-structured cognitive interviews were conducted with 9 urban and rural ALHIV. Three main themes emerged: 1) participants spoke about experiences of HIV stigma specific to a Southern African context, such as anticipating stigma from community members due to ‘punishment from God or ancestors’; 2) participants’ responses uncovered discrepancies between what the items intended to capture and how they understood them and 3) participants’ interpretation of wording uncovered redundant items. Items were revised or removed in consultation with participants. Secondly, we psychometrically assessed and validated this adapted ALHIV stigma scale (ALHIV-SS). We used total population sampling in 53 public healthcare facilities with community tracing. 721 ALHIV who were fully aware of their status were identified and interviewed for the psychometric assessment. Confirmatory factor analysis confirmed a 3-factor structure of enacted, anticipated and internalized stigma. The removal of 3 items resulted in a significant improvement in model fit (*Chi* ^*2*^ *(df)* = 189.83 (33), *p* < .001) and the restricted model fitted the data well (RMSEA = .017; CFI/TLI = .985/.980; SRMR = .032). Standardized factor loadings of indicators onto the latent variable were acceptable for all three measures (.41–.96). Concurrent criterion validity confirmed hypothesized relationships. Enacted stigma was associated with higher AIDS symptomatology (*r* = .146, *p* < .01) and depression (*r* = .092, *p* < .01). Internalized stigma was correlated with higher depression (*r* = .340, *p* < .01), higher AIDS symptomatology (*r* = .228, *p* < .01) and low social support (*r* = −.265, *p* < .01). Anticipated stigma was associated with higher depression (*r* = .203, *p* < .01) and lower social support (*r* = −.142, *p* < .01). The resulting ALHIV-SS has 10 items capturing all three HIV stigma mechanisms experienced by ALHIV. ALHIV-SS will be valuable for evaluating rates and types of stigma, as well as effectiveness of stigma-reduction interventions among ALHIV in Southern Africa.

## Introduction

Sub-Saharan Africa is home to 90 % of the world’s adolescents living with HIV (ALHIV), among whom AIDS-related mortality is on the rise (WHO 2014). HIV stigma can compromise the survival of these youth and facilitate onward HIV transmission by undermining adherence to antiretroviral treatment (ART) (Katz et al. 2013; Rintamaki et al. 2006; Sayles et al. 2009; Susan et al. 2012). HIV stigma and the resultant fear of being identified as HIV-positive may keep adolescents from accessing key health services such as prevention of mother-to-child transmission, HIV testing and ART initiation (Mahajan et al. 2010; Nyblade et al. 2009; Turan and Nyblade 2013). Longitudinal evidence from South Africa suggests that HIV-related stigma has enduring, damaging effects on the mental health of adolescents (Boyes and Cluver 2013), which may further reduce capacity to practice safe sex (Cluver et al. 2013; Meade and Sikkema 2005) and adhere to treatment (Sayles et al. 2009). However, to our knowledge, no measurement tools for assessing HIV stigma among Southern African ALHIV exist (Earnshaw and Chaudoir 2009; Stangl et al. 2013; Stevelink et al. 2012).

Stigma is defined as a process by which individuals are “disqualified from full social acceptance” due to possessing physical, health or behavioral attributes that are deemed “deeply discrediting” (Goffman 1968)*.* The HIV stigma framework specifies three distinct mechanisms through which HIV-positive individuals experience stigma: enacted, anticipated and internalized stigma (Earnshaw and Chaudoir 2009). Enacted stigma refers to experiences of discrimination or having been treated differently due to one’s HIV status. Anticipated stigma refers to the extent to which HIV-positive people perceive or anticipate prejudice against themselves. Internalised stigma occurs when an HIV-positive person endorses negative attitudes associated with HIV and accepts them as applicable to his or her self (Earnshaw et al. 2013).

These three stigma mechanisms may develop independently of one another. For example, when a person is diagnosed with HIV she might decide not to disclose her status to others due to anticipated stigma (Derlega et al. 2004). This would make her susceptible to internalized HIV stigma but less so to enacted stigma. In line with this, HIV-positive respondents in Lesotho, Malawi, South Africa, Swaziland, Tanzania, Kenya, Burkina Faso and Uganda have consistently reported higher levels of internalized than enacted stigma (Cuca et al. 2012; Holzemer et al. 2007; Neuman and Obermeyer 2013). Earnshaw and colleagues have stressed that “by differentiating between HIV stigma mechanisms, researchers may gain a more nuanced understanding of how HIV stigma impacts health and well-being and better inform targeted interventions to improve specific outcomes among people living with HIV” (Earnshaw et al. 2013).

A recent systematic review found no stigma reduction interventions targeting HIV-positive adolescents or children in Sub-Saharan Africa (Stevelink et al. 2012), and one of the reasons for this may be the lack of a culturally-relevant, age appropriate and validated tool for measuring HIV stigma among this population (Earnshaw and Chaudoir 2009; McAteer et al. 2016; Stevelink et al. 2012). Most evaluated interventions provided information and skills-building sessions for non-infected individuals, or attempted to reduce fear of HIV infection through casual contact with key populations (Stangl et al. 2013). Out of 48 stigma reduction interventions, only 3 aimed to reduce manifestations of stigma among HIV-positive individuals in Sub-Saharan Africa (Neema et al. 2012; Tshabalala and Visser 2011; Uys et al. 2009) and none targeted HIV-positive adolescents or children in the region. Measuring HIV stigma as experienced by ALHIV and including indicators such as shame and anticipation of being stigmatized (Fortenberry et al. 2002), would be key for stigma-reduction interventions aiming to impact health-seeking behaviours in this high-risk group.

### Existing HIV Stigma Scales for People Living with HIV

To date, no HIV stigma measures have been validated for use with HIV-infected children or adolescents in Sub-Saharan Africa (McAteer et al. 2016). Systematic review evidence suggests that globally 12 scales are available for measuring HIV stigma among HIV-positive people (Earnshaw and Chaudoir 2009; Stevelink et al. 2012). Of the 12 measures, three were developed in Sub-Saharan Africa (Holzemer et al. 2007; Kalichman et al. 2009; Visser et al. 2008) and none were designed for ALHIV. Holzemer developed a 33-item multi-dimensional stigma scale that was validated in a sample of 1477 HIV-positive adults in Lesotho, Malawi, South Africa, Swaziland and Tanzania (Holzemer et al. 2007). The scale captures internalized stigma and five dimensions of enacted stigma. Kalichman and colleagues’ Internalized AIDS-Related Stigma Scale showed good reliability in South African, Swaziland and the US samples of HIV-positive adults (Kalichman et al. 2009). Visser and colleagues developed two parallel scales, one for measuring stigma among general community members and one for measuring stigma among HIV-positive adult women (Visser et al. 2008). The latter scale measured internalized and anticipated stigma but not enacted stigma (Earnshaw et al. 2013; Visser et al. 2008). These three African stigma scales were pioneering in HIV stigma research in the region. Unfortunately, they were not developed for use with adolescents and none of them capture all three HIV stigma mechanisms.

We conducted a two-stage study in South Africa to address these gaps. The first stage used qualitative methods to cross-culturally adapt an HIV stigma scale previously used with ALHIV in the US (Wright et al. 2007). The second stage psychometrically assessed and validated the adapted ALHIV stigma scale (ALHIV-SS) within the world’s largest survey of HIV-positive adolescents. Ethical approval was provided by Research Ethics Committees at the Universities of Oxford (SSD/CUREC2/12–21) and Cape Town (CSSR 2013/4), Eastern Cape Departments of Health and Basic Education, and ethical review boards of participating hospitals.

## Stage 1: Qualitative Cross-Cultural Adaptation

### Method: Cognitive Interviews

We used Wright and colleagues’ abbreviated version of the Berger stigma scale previously used with ALHIV in the US (Wright et al. 2007). To our knowledge, this was the only HIV stigma scale that measured all three HIV stigma mechanisms among ALHIV. Items were translated and back translated independently by different Xhosa and English-speaking research assistants. Due to the sensitive nature of the questions and in order to reduce social desirability bias, vignettes were added. For example, prior to internalized stigma items a vignette was inserted: “This is Lundi. Living with HIV is difficult for him sometimes. Some days Lundi struggles to feel good about himself. Could you say how much these things are true for you?” (Table 1).

**Table 1:** Stigma items and vignettes used in the cognitive interviews

Internalised stigma vignette and items: This is Lundi. Living with HIV is difficult for him sometimes. Some days Lundi struggles to feel good about himself. Could you say how much these things are true for you? (Nosizi for female respondents)
1. I am very careful who I tell that I have HIV.
2. I worry that people who know I have HIV will tell others.
3. I feel that I am not as good as other kids because I have HIV.
4. Having HIV makes me feel unclean/dirty.
5. Having HIV makes me feel that I’m a bad person.
Anticipated stigma vignette and items: Could you tell us a little bit about what people in your community think about HIV?
1. Most people think that a person with HIV is disgusting.
2. Most people with HIV are rejected when others find out.
Enacted stigma vignette and items: Remember Lundi? He is having a hard time because of his HIV status. Sometimes people treat Lundi differently from other kids just because he is HIV-positive. This is not fair. Could you say how much these things have been true for you? (Nosizi for female respondents)
1. I have been hurt by how people reacted when they learnt about my HIV status.
2. I have stopped socializing with some kids because of their reactions to my HIV status
3. I have lost friends by telling them I have HIV.

Response options: never, sometimes, most of the time

Cross-cultural adaptation of this scale was conducted via one-on-one semi-structured cognitive interviews with 4 rural and 5 urban ALHIV in the Eastern Cape, South Africa. Respondents were recruited from peri-urban and rural areas (age range: 10–19, mean age: 15.6). Voluntary written informed consent was obtained from caregivers and adolescents for a 60-min interview including breaks and games. No incentives were provided, but all adolescents were given certificates and lunch. Cognitive interviewing is a method that is commonly used to uncover inconsistencies between what the measurement items are meant to ask and the way in which members of the target population interpret items (De Silva et al. 2006). If not detected and addressed, such inconsistencies can introduce bias into conclusions drawn from empirical data. Cognitive interviewing involves probing of respondents to interpret the meaning of items and specific terms within the items.

When needed, interpretation was provided by a bilingual research assistant who was trained in qualitative research with ALHIV. Respondents were regularly reminded that they were not expected to respond to the items and that their primary role in the study was to help make the items clearer, easier to respond to and more adolescent-friendly. Respondents were also informed that the interviewers did not design the scale so as to eliminate possible inhibition from suggesting improvements. Respondents were probed to: (1) Read out loud each vignette and each question; (2) Paraphrase the vignette/question in their own words; (3) Provide an example of the concepts mentioned in the vignettes/questions (i.e. ‘Could you tell me an example of when Lundi struggles to feel good about himself?’); (4) Tell the research team ‘How difficult or easy would it be to respond to this question?’; and (5) Where appropriate, propose alternative wording for the vignette/item (Table 1).

### Analysis of Cognitive Interview Data

Data were reviewed using thematic analysis to identify evidence of problems with vignettes, items, and response options. Codes were assigned to summarize and describe responses. These codes were entered into a summary table and listed under the respective item, vignette or response options. Codes were grouped into themes after each interview. A theme consisted of agreement in the codes from two or more participants. Measure adaptations were initiated once a theme emerged from the codes, and draft revisions were used in subsequent rounds of interviews with new participants.

### Cognitive Interview Results

#### HIV Stigma Mechanisms According to South African ALHIV

The cognitive interviews elicited common types of enacted, anticipated and internalized stigma relevant to ALHIV in South Africa that had not been captured in the original measurement used in the US. Findings informed adaptations to the scale prior to psychometric assessment; the adapted items are presented in Table 2.

**Table 2:** Variable names and items generated through cognitive interviews and used in the psychometric assessment

Stigma construct	Observed variables/ indicators/ item wording	Response options
Vignette preceding anticipated stigma items: ‘Could you tell us a little bit about what people in your community think about HIV?’		
Anticipated stigma	‘People in my community think that a person with HIV is disgusting.’	Scale (3-point likert)
	‘People in my community think that HIV is a punishment from God or from ancestors.’	Scale (3-point likert)
Vignette preceding enacted stigma items: ‘Lundi is having a hard time because of his HIV status. Sometimes people treat Lundi differently from other kids just because he is HIV-positive. This is not fair. Could you say how much these things have been true for you in the past year?’ [Nosizi for girls]		
Enacted stigma	‘I have been hurt by how people reacted when they found out I have HIV’^a^	Scale (3-point likert)
	‘I have stopped spending time with some kids because of their reactions to my HIV status.’	Scale (3-point likert)
	‘I have lost friends by telling them I have HIV.’	Scale (3-point likert)
	‘I’ve been teased because of my HIV’	Scale (3-point likert)
Vignette preceding internalised stigma items: ‘This is Lundi [Nosizi for girls]. Living with HIV is difficult for him sometimes. Some days Lundi feels ashamed and he struggles to feel good about himself. Could you say how much these things have been true for you in the past year?’		
Internalized stigma	‘Lundi is very careful who he tells he has HIV. Are you careful who you tell?’^a^	Scale (3-point likert)
	‘Sometimes Lundi feels that he/she is not as good as other kids because he has HIV. Do you ever feel this way?’	Scale (3-point likert)
	‘Sometimes Lundi feels like he/she would rather die than live with HIV. Do you ever feel this way?’	Scale (3-point likert)
	‘Sometimes Lundi feels like he/she is a bad person because he has HIV. Do you ever feel this way?’	Scale (3-point likert)
	‘Sometimes Lundi feels ashamed that he is HIV-positive. Do you ever feel this way?’	Scale (3-point likert)
	‘Sometimes Lundi feels that it is his/her fault that he is HIV-positive. Do you ever feel this way?’^a^	Scale (3-point likert)
	‘Sometimes having HIV makes Lundi feels contaminated and dirty inside. Do you ever feel this way?’	Scale (3-point likert)

^a^Item later deleted due to poor factor loading in the confirmatory factor analysis

##### Enacted Stigma

In open-ended discussions of the vignette describing Lundi, an ALHIV who is ‘treated differently’ because of his HIV status, respondents repeatedly provided examples of being teased. This was in line with previous research with AIDS-affected adolescents in South Africa (Boyes et al. 2013) and items measuring this were included prior to the psychometric assessment.

##### Internalised Stigma

When probed to provide examples of what Lundi experiences when he ‘struggles to feel good about himself’ because of his HIV status, respondents provided examples of shame, guilt and suicidality. One respondent said ‘He is shy to walk on the street because people will point fingers’ and many suggested that Lundi might feel like HIV infection was his fault. Participants also spoke about suicidal ideation and attempts among adolescents who struggle to accept seropositivity. They provided examples of ALHIV ending their life by purposefully defaulting from ART, as well as attempting to overdose from ART. Feelings of shame, guilt and suicidality have already been captured in tools measuring internalized stigma among South African HIV-positive adults (Holzemer et al. 2007; Kalichman et al. 2009; Visser et al. 2008). Items were adapted from existing measurements in consultation with participants (Table 2).

##### Anticipated Stigma

When probed to provide examples of stigma, respondents spoke about people in the community believing that ALHIV have been punished with HIV for bad behaviour. Respondents anticipated being judged for ‘bad behaviours’ if they were sexually active, poor or unable to attend school, for example: ‘If I don’t go to school they will say I am bad and this is why I have HIV’, ‘They will say I deserve this because I am poor’. Punishment was often thought to come from God or from ancestors. This finding coincides with earlier community-based studies in South Africa (Kalichman and Simbayi 2004). We therefore included an item on this in consultation with participants (Table 2).

#### Vignette Utility

Respondents found the vignettes to provide a sense of comfort in knowing that other youth experience HIV stigma as well. In four instances, respondents were confused about internalised stigma questions being phrased in first person (see Table 1). Even though they acknowledged that the given examples of internalized stigma commonly happened to ALHIV, two respondents felt that the original items suggested that they *should* feel this way. They suggested that all internalized stigma items refer to Lundi, the character from the vignette and we made adaptations accordingly, for example: ‘Sometimes Lundi feels that he/she is not as good as other kids because he has HIV. Do you ever feel this way?’ (Table 2).

#### Redundancy

Two items were eliminated because repetitive interpretations were provided when participants were asked to paraphrase items. The two anticipated stigma items originally provided in the US measurement (‘Most people think that a person with HIV is disgusting’ and ‘Most people with HIV are rejected when others find out’) were interpreted similarly. Therefore we retained the item that was easier to interpret by participants (Table 2). Similarly, the two items about disclosure (‘I am very careful who I tell that I have HIV’ and ‘I worry that people who know I have HIV will tell others’) overlapped in terms of the respondents’ comprehension. Here too, only the item that was found easier to interpret by adolescents was retained (Table 2).

**Table 3:** Sample characteristics (*n* = 721)

	Mean (SD) or N (%)
Age	14.65 (2.75)
Female	408 (56.6)
Rural household	137 (19.0)
Child-focused grant recipient	588 (81.6)
Place of interview	
Participant’s home	602 (84.0)
Healthcare facility	85 (11.8)
Other (i.e. school, community centre	41 (5.7)

#### Linguistic Adaptation

Participants’ interpretation of items and specific words within items detected areas for improvement of wording. For example, in the original item on internalized stigma ‘*Having HIV makes me feel unclean or dirty*’, the words dirty and unclean were both interpreted as ‘not having showered’. These words were substituted for ‘contaminated or dirty inside’ to capture subjective feelings of uncleanliness documented through qualitative work on internalised HIV stigma (Lawless et al. 1996). Also, the phrase ‘when they learned about my HIV status’ was understood as reading about one’s HIV status ‘in a book’. This phrase was changed to the Xhosa equivalent of ‘when they found out that I have HIV’ based on consultation with participants.

## Stage 2: Psychometric Assessment of the Adapted ALHIV Stigma Scale (ALHIV-SS)

### Methods: Quantitative Survey and Psychometric Assessment

As part of a larger study on ART adherence among ALHIV, we used total population sampling in public healthcare facilities with community tracing in a mixed urban, peri-urban and rural health district of the Eastern Cape, South Africa. From 2014 to 2015, all public health facilities that provided ART to 5 or more adolescents (aged 10–19) were identified (*n* = 53). Within these facilities, all adolescents who had ever initiated ART were identified through patient files and computerized records (*n* = 1176) and their addresses were recorded for community-tracing purposes. Adolescents were met in the facilities or followed up in their homes so as to ensure inclusion regardless of clinic attendance, treatment defaulting or being lost-to-follow-up. None of the participants from Stage 1 were included in the quantitative stage of the study.

90.1 % (*n* = 1060) of the eligible sample was interviewed. Of the remainder, 4.1 % refused participation (either adolescent or caregiver), 0.9 % had such severe cognitive disability that they were unable to participate, 1.2 % were unable to be interviewed for safety reasons (such as those living in gang homes) and 3.7 % were unable to be traced. Because of the explicit mention of HIV in the stigma scale, only the subsample of ALHIV who were fully aware of their status were asked HIV stigma questions (*n* = 721, 67.7 %) and were included in the present study.

Voluntary informed consent was obtained from caregivers and adolescents for a 90-min interview. No incentives were provided, but all adolescents were given a certificate, snack, toothbrush and toothpaste. So as to prevent inadvertent disclosure of HIV status to community and family members, and to reduce stigma associated with participation in the study, the research was presented in communities as focusing on general needs of adolescents using social and health services, and 467 additional adolescents who were co-resident but HIV-negative, or who lived in neighbouring homes, were also interviewed (not included in these analyses).

Questionnaires were translated and back translated into isi-Xhosa and used mobile-assisted self-interview technology on tablets. Xhosa, English and Afrikaans-speaking interviewers, trained in working with HIV-affected adolescents, read questions in case of low literacy levels or cognitive delay. Confidentiality was maintained, except in cases of significant harm or when participants requested assistance. Where participants reported recent abuse, rape, suicidal attempt or other risk of significant harm, referrals were made to child protection and health services, with follow-up support from the research team.

#### Measures

Measures of depressive symptoms, social support and AIDS symptomatology were included to assess external validity of each stigma mechanism. Based on a previous systematic review, internalized stigma was hypothesized to be associated with low social support, more AIDS-related symptoms and poor mental health (Pantelic et al. 2015). We hypothesized negative relationships between anticipated stigma and social support and mental health based on consistent associations found in previous research (Earnshaw and Chaudoir 2009). We hypothesized enacted stigma to be associated with higher AIDS symptomatology and poor mental health (Earnshaw and Chaudoir 2009; Smith et al. 2008).

*Depressive symptoms* were measured via the Child Depression Inventory short form (CDI-S), which has comparable results with the full CDI (Kovacs 1995). CDI-S has been used with AIDS-affected adolescents in South Africa, displaying acceptable internal consistency (α = .67–.69) (Cluver et al. 2012). CDI-S also demonstrated acceptable internal consistency in the present sample of ALHIV (α = .62).

*Social support* was measured using 9 tangible and emotional support items from the Medical Outcome Study (MOS) Social Support Survey (Sherbourne and Stewart 1991). Items included “How often do you have someone to take you to the doctor if you needed it?” and “How often do you have someone to give you good advice about a crisis?”. Responses were offered on a 3-point likert scale (0:‘Never’; 1:‘Sometimes’; 2:‘Always’). The scale demonstrated strong internal consistency in the present sample of ALHIV (α = .85).

*AIDS symptomatology* was measured via response to the 16-item verbal autopsy, a questionnaire developed to identify symptoms of AIDS in areas with over 20 % HIV prevalence and where data on cause of illness are unavailable or unreliable. Verbal Autopsy is increasingly being used for determining AIDS mortality in generalized epidemics (Cluver et al. 2012; Doctor and Weinreb 2003; Hosegood et al. 2004) and recent research found the method to have 75–83 % sensitivity and 74–79 % specificity among adult subjects (Lopman et al. 2010). Items included ‘asthma, lung problems and trouble breathing for more than two days’, ‘sores in the mouth, hands and feet, parts of the body’, and ‘diarrhoea or runny tummy for more than two days’. Responses were offered on a 3-point scale (0:‘Never’; 1:‘Sometimes’; 2:‘Most of the time’).

Age, gender, rural household location, receipt of child-focused welfare grants and place of interview were recorded for descriptive purposes. *Receipt of child-focused welfare grants* was assessed via participant response to ‘How many child support grants does your household receive?’ and ‘How many foster care grants does your household receive?’. Responses were recoded as a dichotomous variable to determine household receipt of any child-focused grant (0: no access to child-focused grants; 1: access to one or more child-focused grants at the household level). *Place of interview* was recorded by the research assistant at the start of the interview. The tablet offered the following options: participant’s home, clinic, hospital, school, church, community centre and other. This was later recoded into participant’s home, healthcare facility, school and other.

### Psychometric Assessment Analysis

All psychometric assessment analyses were conducted within the subsample of ALHIV who were aware of their status (*n* = 721) using MPlus7. Confirmatory factor analysis tested whether ALHIV-SS consisted of the three hypothesized factors: anticipated, enacted and internalised stigma. Items loading below .4 were excluded from the scale (Bowen and Guo 2012). Model fit was assessed via multiple goodness-of-fit measures. Comparative Fit Index (CFI) and Tucker Lewis Index (TLI) above .95 (Bentler 1990; Hu and Bentler 1995), and Root Mean Square Error of Approximation (RMSEA) and standardized root mean-square residual (SRMR) values below .05 indicated good model fit (Bowen and Guo 2012). *χ* ^2^ is not recommended for assessing goodness-of-fit in large samples as it is sensitive to sample size and is prone to Type 2 error (Schermelleh-Engel et al. 2003; Vandenberg 2006). *χ* ^2^ was therefore only noted to compute changes in *χ* ^2^ and assess improvement in model fit after modification (Δχ^*2*^). Internal consistency (Cronbach’s α) assessed reliability. As detailed in the measures section of this paper, concurrent criterion validity was assessed through associations between HIV stigma constructs and correlates identified in earlier reviews and meta-analyses of quantitative research.

### Psychometric Assessment Results

Table 3 reports socio-demographic characteristics of the sample of ALHIV who were fully aware of their status (*n* = 721). The mean age was 14.6 (SD = 2.75). 56.6 % of the sample was female and 19 % lived in rural areas. 81.6 % of the sample received a child-focused grant, indicative of relative material deprivation in the sample. The majority of participants were interviewed in their home (84 %), 11.8 % were interviewed in clinics or hospitals and 5.7 % were interviewed in other spaces such as their schools or community centres.

#### Confirmatory Factor Analysis (CFA) Results

Table 4 summarizes item phrasing, response options and frequencies for indicators that were included in the CFA. So as to account for non-normal data, CFA was run on a 3-factor robust maximum likelihood (MLR) model. Enacted stigma items were constrained to load onto the enacted stigma factor; anticipated stigma items were constrained to load onto the anticipated stigma factor; and internalized stigma items were constrained to load onto the internalized stigma factor.

**Table 4:** Response option frequencies for each stigma item (*n* = 721)

Stigma construct	Indicator	N (%)		
		Never	Sometimes	Most of the time
Anticipated stigma	‘People in my community think that a person with HIV is disgusting.’	520 (72.1)	158 (21.9)	43 (6.0)
	‘People in my community think that HIV is a punishment from God or from ancestors.’	578 (80.2)	115 (16.0)	28 (3.9)
Enacted stigma	‘I have been hurt by how people reacted when they found out I have HIV’^a^	663 (92.0)	45 (6.2)	13 (1.8)
	‘I have stopped spending time with some kids because of their reactions to my HIV status.’	698 (96.8)	19 (2.6)	4 (0.6)
	‘I have lost friends by telling them I have HIV.’	706 (97.9)	13 (1.8)	2 (0.3)
	‘I’ve been teased because of my HIV’	702 (97.4)	14 (1.9)	5 (0.7)
Internalized stigma	‘Lundi is very careful who he tells he has HIV. Are you careful who you tell?’^a^	145 (20.1)	81 (11.2)	480 (66.6)
	‘Sometimes Lundi feels that he/she is not as good as other kids because he has HIV. Do you ever feel this way?’	596 (82.7)	87 (12.1)	23 (3.2)
	‘Sometimes Lundi feels like he/she would rather die than live with HIV. Do you ever feel this way?’	652 (90.4)	49 (6.8)	5 (0.7)
	‘Sometimes Lundi feels like he/she is a bad person because he has HIV. Do you ever feel this way?’	661 (91.7)	43 (6.0)	2 (0.3)
	‘Sometimes Lundi feels ashamed that he is HIV-positive. Do you ever feel this way?’	605 (83.9)	90 (12.5)	11 (1.5)
	‘Sometimes Lundi feels that it is his/her fault that he is HIV-positive. Do you ever feel this way?’^a^	644 (89.3)	51 (7.1)	11 (1.5)
	‘Sometimes having HIV makes Lundi feels contaminated and dirty inside. Do you ever feel this way?’	657 (91.1)	45 (6.2)	4 (0.6)

^a^Item later deleted due to poor factor loading in the confirmatory factor analysis

Results of the full model CFA are presented in Table 5. Fit indices indicated that the model fitted the data well (RMSEA = .016; CFI/TLI = .983/.978; SRMR = .038). However, factor loadings ranged between .39–.96 (Table 5), with three items failing to meet the pre-specified loading cutoff of .40 (Bowen and Guo 2012). Further inspection of wording confirmed that these items were ambiguous or vague in relation to the intended theoretical stigma constructs. For example one of the items was ‘I have been hurt by how people reacted when they found out I have HIV’. By asking about other people’s behaviors as well as the respondent’s subjective response to these behaviors, this item taps into both enacted and internalized stigma constructs. Such items were removed from further analysis and CFA was rerun on the restricted model.

**Table 5:** Results of first CFA, full model

Stigma domain:	Anticipated		Enacted		Internalised	
Item:	*β*	*SE*	*β*	*SE*	*β*	*SE*
Participant thinks that people in community think HIV+ people are disgusting	.957***	.115				
Participant thinks that people in community think HIV is a punishment from God or ancestors	.567***	.076				
Participant has been teased because of HIV status			.643***	.114		
Participant has been hurt by people’s reactions to their HIV status			.388***	.070		
Participant has stopped spending time with some kids because of his/her HIV status			.605***	.118		
Participant has lost friends because of his/her HIV status			.409*	.167		
Participant is ashamed of their HIV status					.639***	.052
Participant feels they aren’t as good as other kids because of HIV status					.608***	.055
Participant feels that they would rather die than be living with HIV					.622***	.061
Participant feels like a bad person because of HIV					.657***	062
Participant feels that HIV is their fault					.390***	.078
Participant feels that HIV makes them dirty inside					.653***	.066
Participant is very careful who they tell about their status					.102**	.034

Model fit: RMSEA = .016; CFI/TLI = .983/.978; *χ* ^2^ (df) = 705.787(78)***; SRMR = .038

When CFA was run on the restricted model (Table 6), fit indices indicated that it fitted the data well (RMSEA = .017; CFI/TLI = .985/.980; SRMR = .032). The removal of 3 items resulted in a significant improvement in model fit (Δχ^*2*^ *(df)* = 189.83 (33), *p* < .001). Overall, standardized factor loadings of indicators onto the latent variable were acceptable for all three measures, ranging between .57–.96 for anticipated stigma, .41–.68 for enacted stigma and .62–.65 for internalized stigma. Latent correlations between internalised stigma and anticipated (*r* = .239, *p* < .01) and enacted stigma (*r =* .092, *p* < .01) were significant with weak effect sizes. Anticipated and enacted stigma were not significantly correlated (r = .117). Additional modifications to the measurement model were not carried out due to the very good fit of the model.

**Table 6:** Results of restricted model CFA

Stigma mechanism:	Anticipated		Enacted		Internalised	
Item:	*β*	*SE*	*β*	*SE*	*β*	*SE*
Participant thinks that people in community think HIV+ people are disgusting	.961***	.122				
Participant thinks that people in community think HIV is a punishment from God or ancestors	.565***	.079				
Participant has been teased because of HIV status			.681***	.098		
Participant stopped spending time with kids because of his/her HIV status			.577***	.133		
Participant has lost friends because of his/her HIV status			.412*	.173		
Participant is ashamed of their HIV status					.647***	.053
Participant feels they aren’t as good as other kids because of HIV status					.618***	.055
Participant feels that they would rather die than be living with HIV					.624***	.061
Participant feels like a bad person because of HIV					.646***	064
Participant feels that HIV makes them dirty inside					.646***	.066

Model fit: RMSEA = .017; CFI/TLI = .985/.980; *χ* ^2^ (df) = 515.957 (45)***; SRMR = .032

Improvement in model fit: Δχ^2^ (df) = 189.83 (33)***

The final ALHIV-SS resulted in 10 items: 2 anticipated, 3 enacted and 5 internalized stigma items. Internal consistency / Cronbach’s α levels were .70, .57 and .75 for anticipated, enacted and internalized stigma respectively.

#### Concurrent Criterion Validity

Correlations testing concurrent criterion validity confirmed hypothesized relationships. Enacted stigma was associated with higher AIDS symptomatology (*r* = .146, *p* < .01) and depression (*r* = .092, *p* < .01). Internalized stigma was correlated with depression (*r* = .340, *p* < .01), AIDS symptomatology (*r* = .228, *p* < .01) and low social support (*r* = −.265, *p* < .01). Anticipated stigma was associated with depression (*r* = .203, *p* < .01) and low social support (*r* = −.142, *p* < .01).

## Discussion

This paper provides a comprehensive report of the qualitative and quantitative adaptation process of an ALHIV stigma scale from the US to the South African context. Two linked stages were presented. The first stage used cognitive interviews to cross-culturally adapt an HIV stigma scale previously used with ALHIV in the US (Wright et al. 2007). The second stage conducted a psychometric assessment and validation of the adapted ALHIV stigma scale (ALHIV-SS) in a representative sample of 721 HIV-positive adolescents who were aware of their status.

The resulting ALHIV-SS has 10 items and measures all three HIV stigma mechanisms experienced by ALHIV: enacted, anticipated and internalized stigma. To our knowledge, this is the first HIV stigma measurement to measure all three HIV stigma mechanisms within an HIV-positive sample in Sub-Saharan Africa. This is also the first HIV stigma tool specifically designed for and in collaboration with ALHIV in the region. The mixed-methods approach to the scale adaptation has minimized potential bias for future empirical research utilizing ALHIV-SS. For example, cognitive interviews uncovered ambiguities in Xhosa wording that standard translation and back translations did not detect. On the other hand, by identifying items with poor factor loadings, the psychometric assessment helped recognize and remove theoretically ambiguous items in the measurement.

ALHIV-SS was validated within a large sample of ALHIV, of which only 11.8 % were interviewed in healthcare facilities. The community tracing used in this study is likely to have generated a more representative sample of ALHIV than would have been the case with a sample of ALHIV who actively access services. To our knowledge, previous quantitative studies on HIV stigma among HIV-positive individuals recruited through healthcare facilities, community organizations or other service providers (Pantelic et al. 2015; Stangl et al. 2013). Such recruitment approaches may have excluded the most vulnerable ALHIV, whose access to health and other services can be limited due to high anticipated or internalized stigma.

This scale is not without limitations. Firstly, although the consistency of internalised and anticipated stigma subscales were good, the enacted stigma subscale displayed an alpha of 0.57. Given that Cronbach’s alpha is affected by the number of items (only 3 in this subscale), there may be value in devising a longer scale for more detailed research on enacted HIV stigma among adolescents living with HIV. Care should be taken to involve the target population, ALHIV, in the development of such a scale. It should also be noted that shorter questionnaires are essential for reducing research burden for ALHIV, many of whom are cognitively delayed (Sherr et al. 2014). Cronbach’s alpha of the enacted stigma sub-scale might also have been affected by the inter-correlations between items. This problem is not unique to the present scale. Similar measurements of bullying or abuse victimization also commonly display poor internal consistency because the phenomena are not one-dimensional. For example, a recent systematic review of bullying scales found that internal consistency of included measures ranged between α = 0.25 and α = 0.96 (Vivolo-Kantor et al. 2014).

Secondly, while the scale showed good psychometric properties, it is important to note that it was cross-culturally adapted and validated within a sample of Xhosa-speaking ALHIV in the Eastern Cape, South Africa. Given that stigma manifestations are culturally and socially embedded, the scale’s generalizability and usability within other Southern African contexts might be limited. Nevertheless, future research with HIV-positive adolescents could benefit from the present scale as a starting point for further adaptations and translations.

In line with previous research, our findings confirm that enacted, anticipated and internalized stigma are independent constructs (Earnshaw and Chaudoir 2009; Earnshaw et al. 2013). But to our knowledge, this is the first time that the relationship between the three HIV stigma mechanisms has been assessed among ALHIV in Southern Africa. Anticipated and enacted stigma factors were not significantly correlated, confirming that the factors measure divergent constructs. There was a statistically significant correlation between internalized and enacted stigma but the strength of this relationship was very close to the line of no effect suggesting that they too are independent constructs. This has important implications for theory and intervention development, which has so far heavily focused on reducing HIV-related prejudice and discrimination among the general public rather than reducing HIV stigma as experienced by HIV-positive individuals. While reducing discriminative behaviors among the general public might reduce experiences of enacted stigma among ALHIV, our findings suggest that enacted stigma occurs independently of anticipated and internalized stigma. Therefore more interventions aiming to reduce anticipated and internalized stigma are urgently needed.

The ALHIV-SS will be valuable for evaluating rates and types of stigma, and effectiveness of interventions aiming to reduce HIV stigma among ALHIV in Southern Africa. Such interventions are urgently needed: between 2005 and 2012 there has been a 50 % increase in reported AIDS-related deaths among ALHIV compared with the 30 % decline seen in the general population (WHO 2014). This alarming trend has been attributed to “poor prioritization of adolescents in national HIV plans, inadequate provision of accessible and acceptable HIV testing and counseling and treatment services and lack of support for adolescents to remain in care and adhere to [life-saving] ART” (WHO 2013). World Health Organization recommendations for policy makers and program managers frequently cite stigma as a key barrier to service access and utilization among ALHIV (WHO 2013). But to our knowledge, no well-established HIV stigma reduction interventions exist for Southern African ALHIV (Stangl et al. 2013). A validated HIV stigma measure such as ALHIV-SS, designed specifically for this population, was a prerequisite for developing such interventions and assessing their effectiveness.

## References

[CR1] Bentler PM (1990). Comparative fit indexes in structural models. Psychological Bulletin.

[CR2] Bowen N, Guo S (2012). Structural equation modeling.

[CR3] Boyes M, Cluver LD (2013). Relationships among HIV/AIDS orphanhood, stigma, and symptoms of anxiety and depression in south African youth: a longitudinal investigation using a path analysis framework. Clinical Psychological Science.

[CR4] Boyes, M.E., Mason, S.J., Cluver, L.D. (2013). Validation of a brief stigma-by-association scale for use with HIV/AIDS-affected youth in South Africa. *AIDS Care*, *25*(2), 215–222. http://ovidsp.ovid.com/ovidweb.cgi?T=JS&PAGE=reference&D=medl&NEWS=N&AN=22774842. 10.1080/09540121.2012.699668 22774842

[CR5] Cluver L, Orkin M, Boyes ME, Gardner F, Nikelo J (2012). AIDS-orphanhood and caregiver HIV/AIDS sickness status: effects on psychological symptoms in South African youth. Journal of Pediatric Psychology.

[CR6] Cluver, L., Orkin, M., Boyes, M.E., Sherr, L., Makasi, D., Nikelo, J. (2013). Pathways from parental AIDS to child psychological, educational and sexual risk: developing an empirically-based interactive theoretical model. *Social Science & Medicine (1982)*, *87*, 185–193. http://ovidsp.ovid.com/ovidweb.cgi?T=JS&PAGE=reference&D=medl&NEWS=N&AN=23631794. 10.1016/j.socscimed.2013.03.028 23631794

[CR7] Cuca YP, Onono M, Bukusi E, Turan JM (2012). Factors associated with pregnant women’s anticipations and experiences of HIV-related stigma in rural Kenya. AIDS Care.

[CR8] De Silva MJ, Harpham T, Tuan T, Bartolini R, Penny ME, Huttly SR (2006). Psychometric and cognitive validation of a social capital measurement tool in Peru and Vietnam. Social Science & Medicine (1982).

[CR9] Derlega V, Winstead B, Greene K, Serovich J, Elwood W (2004). Reasons for HIV disclosure/nondisclosure in close relationships: testing a model of HIV-disclosure decision making. Journal of Social and Clinical Psychology.

[CR10] Doctor HV, Weinreb AA (2003). Estimation of AIDS adult mortality by verbal autopsy in rural Malawi. AIDS.

[CR11] Earnshaw V, Chaudoir SR (2009). From conceptualizing to measuring HIV stigma: a review of HIV stigma mechanism measures. AIDS and Behavior.

[CR12] Earnshaw V, Smith LR, Chaudoir SR, Amico KR, Copenhaver MM (2013). HIV stigma mechanisms and well-being among PLWH: a test of the HIV stigma framework. AIDS and Behavior.

[CR13] Fortenberry JD, McFarlane M, Bleakley A, Bull S, Fishbein M, Grimley DM (2002). Relationships of stigma and shame to gonorrhea and HIV screening. American Journal of Public Health.

[CR14] Goffman E (1968). Stigma: the management of spoiled identity.

[CR15] Holzemer WL, Uys LR, Chirwa ML, Greeff M, Makoae LN, Kohi TW (2007). Validation of the HIV/AIDS Stigma Instrument - PLWA (HASI-P). AIDS Care.

[CR16] Hosegood V, Vanneste A, Timæus IM (2004). Levels and causes of adult mortality in rural South Africa: the impact of AIDS. AIDS.

[CR17] Hu L, Bentler PM (1995). Evaluating model fit. Structural equation modeling: Concepts, issues, and applications.

[CR18] Kalichman SC, Simbayi LC (2004). Traditional beliefs about the cause of AIDS and AIDS-related stigma in South Africa. AIDS Care.

[CR19] Kalichman SC, Simbayi LC, Cloete A, Mthembu PP, Mkhonta RN, Ginindza T (2009). Measuring AIDS stigmas in people living with HIV/AIDS: the Internalized AIDS-Related Stigma Scale. AIDS Care.

[CR20] Katz, I.T., Ryu, A.E., Onuegbu, A.G., Psaros, C., Weiser, S.D., Bangsberg, D.R., & Tsai, A.C. (2013). Impact of HIV-related stigma on treatment adherence: systematic review and meta-synthesis. *Journal of the International AIDS Society*, *16*(3 Suppl 2), 18640. http://www.pubmedcentral.nih.gov/articlerender.fcgi?artid=3833107&tool=pmcentrez&rendertype=abstract. Accessed 3 December 2013. 10.7448/IAS.16.3.18640 PMC3833107 24242258

[CR21] Kovacs, M. (1995). Manual: the children’s depression inventory. In *Multi-Health Systems*. Toronto.

[CR22] Lawless S, Kippax S, Crawford J (1996). Dirty, diseased and undeserving: the positioning of HIV positive women. Social Science and Medicine.

[CR23] Lopman B, Cook A, Smith J, Chawira G, Urassa M, Kumogola Y (2010). Verbal autopsy can consistently measure AIDS mortality: a validation study in Tanzania and Zimbabwe. Journal of Epidemiology and Community Health.

[CR24] Mahajan AP, Sayles JN, Patel VA, Remien RH, Szekeres G, Coates TJ (2010). Stigma in the HIV/AIDS epidemic: a review of the literature and recommendations for the way forward. AIDS.

[CR25] McAteer, C. I., Truong, N.-A. T., Aluoch, J., Deathe, A. R., Nyandiko, W. M., Marete, I., & Vreeman, R. C. (2016). A systematic review of measures of HIV/AIDS stigma in paediatric HIV-infected and HIV-affected populations. *Journal of the International AIDS Society, 19*(1), 21204. doi:10.7448/IAS.19.1.21204. 10.7448/IAS.19.1.21204 PMC5055615 27717409

[CR26] Meade CS, Sikkema KJ (2005). HIV risk behavior among adults with severe mental illness: a systematic review. Clinical Psychology Review.

[CR27] Neema S, Atuyambe LM, Otolok-Tanga E, Twijukye C, Kambugu A, Thayer L, McAdam K (2012). Using a clinic based creativity initiative to reduce HIV related stigma at the Infectious Diseases Institute, Mulago National Referral Hospital, Uganda. African Health Sciences.

[CR28] Neuman M, Obermeyer CM (2013). Experiences of stigma, discrimination, care and support among people living with HIV: a four country study. AIDS and Behavior.

[CR29] Nyblade L, Stangl A, Weiss E, Ashburn K (2009). Combating HIV stigma in health care settings: what works?. Journal of the International AIDS Society.

[CR30] Pantelic M, Shenderovich Y, Cluver L, Boyes M (2015). Predictors of internalised HIV-related stigma: a systematic review of studies in sub-Saharan Africa. Health Psychology Review.

[CR31] Rintamaki LS, Davis TC, Skripkauskas S, Bennett CL, Wolf MS (2006). Social stigma concerns and HIV medication adherence. AIDS Patient Care and STDs.

[CR32] Sayles JN, Wong MD, Kinsler JJ, Martins D, Cunningham WE (2009). The association of stigma with self-reported access to medical care and antiretroviral therapy adherence in persons living with HIV/AIDS. Journal of General Internal Medicine.

[CR33] Schermelleh-Engel K, Moosbrugger H, Müller H (2003). Evaluating the fit of structural equation models: tests of significance and descriptive goodnessof-fit measures. Methods of Psychological Research Online.

[CR34] Sherbourne C, Stewart A (1991). The MOS social support survey. Social Science & Medicine.

[CR35] Sherr L, Croome N, Parra Castaneda K, Bradshaw K, Herrero Romero R (2014). Developmental challenges in HIV infected children—An updated systematic review. Children and Youth Services Review.

[CR36] Smith R, Rossetto K, Peterson BL (2008). A meta-analysis of disclosure of one’s HIV-positive status, stigma and social support. AIDS Care.

[CR37] Stangl, A.L., Lloyd, J.K., Brady, L.M., Holland, C.E., Baral, S. (2013). A systematic review of interventions to reduce HIV-related stigma and discrimination from 2002 to 2013 : how far have we come ? *Journal of the International AIDS So*, *16*. 10.7448/IAS.16.3.18734 PMC3833106 24242268

[CR38] Stevelink SAM, Wu IC, Voorend CGN, Van Brakel WH (2012). The psychometric assessment of internalized stigma instruments : a systematic review. Stigma, Research and Action.

[CR39] Susan LR, Janet R, Senica G, Ross D, Katz JN, Freedberg KA (2012). Depressive symptoms and their impact on health-seeking behaviors in newly-diagnosed HIV-infected patients in Durban, South Africa. AIDS and Behavior.

[CR40] Tshabalala J, Visser M (2011). Developing a cognitive behavioural therapy model to assist women to deal with HIV and stigma. South African Journal of Psychology.

[CR41] Turan, J.M., & Nyblade, L. (2013). HIV-related stigma as a barrier to achievement of global PMTCT and maternal health goals: a review of the evidence. *AIDS and Behavior*, 2528–2539. doi:10.1007/s10461-013-0446-8. 10.1007/s10461-013-0446-8 23474643

[CR42] Uys L, Chirwa M, Kohi T, Greeff M, Naidoo J, Makoae L (2009). Evaluation of a health setting-based stigma intervention in five African countries. AIDS Patient Care and STDs.

[CR43] Vandenberg RJ (2006). Statistical and methodological myths and urban legends. Organizational Research Methods.

[CR44] Visser M, Kershaw T, Makin JD, Forsyth BWC (2008). Development of parallel scales to measure HIV-related stigma. AIDS and Behavior.

[CR45] Vivolo-Kantor A, Martell B, Holland K, Westby R (2014). A systematic review and contenct analysis of bullying and cyber-bullying measurement strategies. Aggression and Violent Behavior.

[CR46] WHO. (2013). *HIV and Adolescents: Guidance for HIV testing and counselling and care for adolescents living with HIV*. Geneva

[CR47] WHO. (2014). *Health for the world’s adolescents*. Geneva.

[CR48] Wright K, Naar-King S, Lam P, Templin T, Frey M (2007). Stigma scale revised: reliability and validity of a brief measure of stigma for HIV+ youth. Journal of Adolescent Health.

